# Bilateral inverted papilloma: case report and literature review

**DOI:** 10.1016/S1808-8694(15)31103-4

**Published:** 2015-10-19

**Authors:** Raquel Salomone, Cícero Matsuyama, Osvaldo Giannotti Filho, Marcia Lanzoni De Alvarenga, Eulógio Emílio Martinez Neto, Adriana Gonzaga Chaves

**Affiliations:** 1 MD. Otorhinolaryngologist Resident - CEMA Hospital; 2 M.S. PhD. Professor of Otorhinolaryngology - UNIFESP/EPM- São Paulo, Otorhinolaryngology Residency Coordinator - CEMA Hospital - São Paulo; 3 PhD. Professor of Pathology - UNIFESP/EPM- São Paulo; 4 Clinical Pathology Resident Physician - UNIFESP/EPM- São Paulo; 5 Otorhinolaryngologist; 6 Otorhinolaryngologist - CEMA Hospital -São Paulo. MSc. Student in Otorhinolaryngology - UNIFESP/EPM- São Paulo

**Keywords:** papilloma, inverted papilloma, computed tomography

## Abstract

The inverted papilloma is an uncommon unilateral nasosinusal benign tumor. The clinical picture presents nonspecific signs and symptoms, such as unilateral nasal obstruction, anosmia and headache. The diagnosis is established by anamnesis, physical exam, computed tomography and magnetic resonance imaging. Treatment is essentially surgical. This report has the objective of presenting an uncommon bilateral inverted nasal papilloma and making a literature review.

## INTRODUCTION

The term papilloma means neoplasia with epithelial growth. The first report of this type of tumor in the nasal cavity was made by Ward et al. in 1854^1^^,^^2^.

The inverted papilloma (IP) is a rare and benign nasosinusal tumor, bearing an incidence of 0.75 to 1.5 cases per 100 thousand inhabitants/year3–8. Representing 0.5 to 4% of all nasal tumors^1^^,^^2^^,^^9^ and from 91 to 99% of the cases are unilateral^7^.

IP originate from the nasal cavity lateral wall, and it secondarily affects the maxillary, ethmoidal, frontal and sphenoid sinuses. The primary involvement of the paranasal sinuses is extremely rare, happening only to 5% of the cases^10^, ^11^, ^12^. The first case of a sphenoidal inverted papilloma was described by John et al. in 2002^7^.

IPs are 4 to 5 times more frequent in males, with greater prevalence in Caucasians, between their 5th and 6th decades of life^8^.

Although benign, the inverted papilloma is characterized by an aggressive growth, great invasion potential^7^^,^^13^^,^^14^, being multicentric (12%), high recurrence rates^1^^,^^2^^,^^9^ and malignization (2 to 53%)^13^. About 10% of he IP cases with cellular atypia are associated with squamous cells carcinoma^8^, ^9^, ^10^, ^11^, ^12^.

Signs and symptoms are unspecific and may cause unilateral nasal obstruction, epistaxis, olfactory disorders and recurrent rhinosinusitis^15^.

Diagnosis is carried out by history taking, otorhinolaryngological exam and image exam. CT scan (CT) and magnetic resonance image (MRI), of the nasal cavities and paranasal sinuses are important to assess the size, extension and anatomical relations of the tumor, and they also help differentiate them from other nasosinusal diseases and also in cases of orbital and/or intracranial complications.

The main differential diagnoses are antrochoanal polyps, nasal cavity squamous polyp, fibrous dysplasia, gigantic cells granuloma and other neoplasia^8^^,^^11^^,^^12^.

Treatment is surgical. Surgical techniques and access must be broadly studied and individualized.

## CASE REPORT

A 51 year-old male complained of nasal obstruction for one year, associated with hyposmia and mucopurulent rhinorrhea. He did not complain of epistaxis, headache or visual impairment.

The otorhinolaryngological exam revealed inferior nasal turbinate moderate hypertrophy and mucosa paleness, with mucopurulent secretion. Oroscopy and otoscopy did not show alterations.

Video-nasofibroscopy showed a large amount of mucopurulent secretion, associated with a lobulated and polypoid tumor, of rough surface and firm, occupying the lateral wall of both nasal cavities.

**Figure 1:**
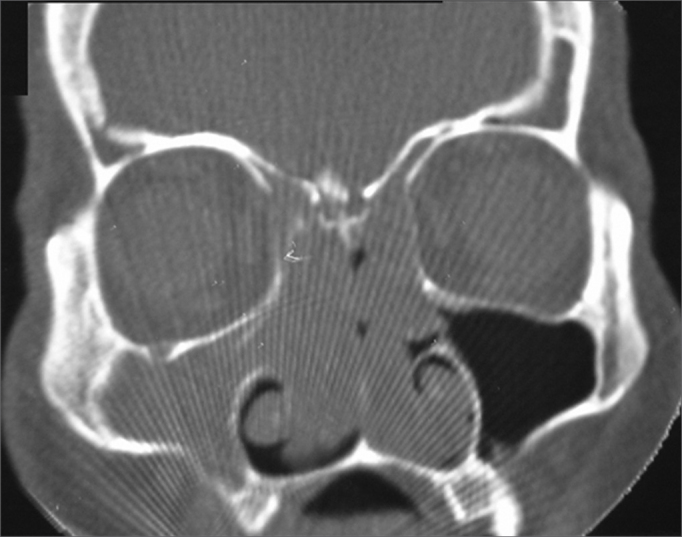
Nasal cavity and paranasal sinuses CT scan. Coronal cross-section, showing the bilateral involvement of nasal cavities, ethmoidal sinuses and right maxillary sinus.

**Figure 2:**
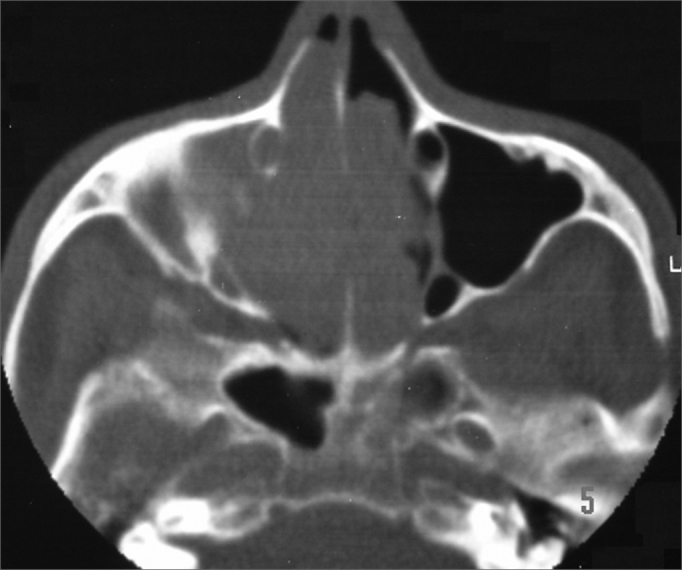
Nasal cavity and paranasal sinuses CT scan. Axial cross-section, showing bilateral involvement of the nasal cavities and right maxillary sinus.

Nasal and paranasal sinuses CT scan revealed a hyperdense image occupying both nasal cavities, ethmoidal, frontal, sphenoid and right maxillary sinuses, enlarging the ostium-meatal complex in both sides and involving the rhino-pharynx. There were no signs of bone lysis (Figure 1, Figure 2).

The patient was then submitted to nasosinusal endoscopic surgery with bilateral total ethmoidectomy, frontal sinusectomy, sphenoidectomy and maxillary antrostomy with tumor extraction in two resection blocks together with most of the lamina papyracea bilaterally.

The pathology exam revealed a bilateral inverted papilloma with no signs of cell atypia. It was carried out in blocks in another institution that confirmed our diagnosis of a bilateral inverted papilloma

Post-operative evolution was satisfactory. Control was carried out every two months in the first 6 months after surgery through nasal endoscopic exam that showed a re-epithelized nasal cavity with no signs of disease recurrence. We then ordered nasal CT scan and MRI, but the patient has not returned yet.

## DISCUSSION

The inverted papilloma, also called Schneider, papilloma, Ewing papilloma, transitional cells papilloma, epithelial papilloma villus cancer, transitional cells benign tumor and papillomatosis, is a benign sinusal tumor with undefined etiology^1^^,^^2^^,^^8^^,^^12^.

The name inverted is justified by the endophilic growth of the superficial epithelium to inside the adjacent stroma^8^^,^^9^^,^^12^. This nasal epithelium proliferates and becomes metaplastic, originating many histological patterns (with internal microcysts) responsible for the high malignancy potential^8^^,^^12^. Ringertz, in 1938^11^, described as IP man papilloma the local invasive growth and epithelial invasion.

The main theory on IP etiology proposes that Schneider membrane, which forms the nasosinusal tract mucosa, originates from the ectodermal invasion of the olfactory placoid. This membrane would then suffer a number of structural changes, causing a greater predisposition for neoplastic differentiation^9^^,^^15^. Other possible etiologies are: inflammatory origin and/or chronic infectious rhinosinusitis, exposure to toxic substances, allergic processes, Epstein-Barr virus and Human Papilloma Virus (HPV) of subtypes 6, 11 and 167,10,11. HPV is associated with the disease pathogenesis, happening in 14% of all inverted papilloma and 100% of the exophytic papillomas^8^.

Histologically, the papillomas can be divided in three types: fungiform or exophytic, which stems from the anterior septum and has the macroscopic aspect of a common wart, the columnar, which stems from the lateral nasal wall and the middle meatus, and the inverted, previously described, with three fold higher chance for malignization if compared to the columnar papilloma^2^^,^^8^.

The clinical aspect of the IP is unilateral nasal obstruction (98%), rhinorrhea (17%), epistaxis (6%), anosmia (4%), headache and frontal pain. The tumor can extend to outside the nasal cavity in 7% of the cases, in 3% of the cases it extends to the nasopharynx and less than 2% to the pterygopalatine and intracranial fossa^1^^,^^2^^,^^8^.

The diagnosis must start by a detailed anamnesis, investigating environmental exposure, noxious habits, allergies and associated diseases, and by complete otorhinolaryngological exam. Endoscopic and radiological (CT and MRI) exams are fundamental for tumor study and diagnosis. Biopsy together with histopathology establish the diagnosis, however must not be carried out before prior histopathology exam in order to rule out the presence of a vascularized tumor (juvenile nasoangiofibroma) or lesions extending to the central nervous system (meningocele and meningoencephalocele)^8^.

Nasal polyps, 25 times more frequent than IPs2, presents respiratory mucosa with stromal edema and eosinophillic infiltrate, and must constitute a differential diagnosis, just like the antrochoanal polyp, squamous polyp, nasal vestibule polyp, fibrous dysplasia, giant cells granuloma and neoplasias^8^^,^^11^^,^^12^.

Recently, Krouse^10^ proposed IP staging in four groups based on tumor invasion to the paranasal sinuses and its possible malignant transformation^7^^,^^10^.

Nasal cavity and paranasal sinuses CT scan suggest IP when there is an image of soft tissue present from the middle meatus all the way to the adjacent maxillary antrum, through an enlarged maxillary ostium,^8^ as the one seen in our patient. Such image may contain areas of hyperdensity (calcifications and/or sclerosis) or deformities on the bone wall of the affected sinus. An antrochoanal tumor with bone deformity (30%) associated with sclerosis suggests slow growth, which is characteristic of PIs^11^^,^^12^.

MRI provides a more accurate assessment of tumor boundaries and implantation site, differentiating it from the adjacent inflammatory tissue and it is also the exam of choice for post-operative follow up^12^. T1 weighed or T1 with fat suppression weighed MRI better assesses the cases of neighboring tissue invasion, such as the orbit, nasopharynx and central nervous system. In T2 weighed, the tumor appears as an intermediate signal and the inflammatory tissue as a hyperintensity signal. Contrast is unable o differentiate IPs from other nasosinusal tumors^8^.

Treatment is surgical. In the past, we used lateral rhinotomy, middle face degloving or medial maxillectomy with tumor en bloc resection. During the 80's, with nasosinusal endoscopic and microendoscopic surgeries, the procedures became less invasive; however, there has been an increase in tumor recurrence rates, counter-indicating the endoscopic approach when performed without the external access^1^^,^^2^^,^^4^^,^^12^. Stankwigcz et al., in 1993^14^, proposed endoscopic surgery only in the cases of unilateral disease without malignancy characteristics, confined to the middle meatus and middle turbinate. Oikawa et al. advocate endoscopic surgery for tumors in stages 1 and 27 (limited to the nasal cavity or ethmoidal sinus, medial and upper maxillary sinus, respectively). Many authors agree on the need of removing bone and adjacent periosteum at disease site, or burr the bone with a diamond drill bit^8^^,^^13^, especially in the area that divides the maxillary roof and the lamina papyracea, where the tumor recurs the most^8^.

Radiotherapy (RT) is controversial. Weissele^9^ and Terance^2^ are only indicated for inoperable benign tumors or multiple recurrent lesions. Jankowski^10^ advocates RT when the inverted papilloma is associated with carcinoma. Atlug^8^, on the other hand, stresses that radiotherapy is inefficient and stresses the risk of such procedure causing tumor malignancy and osteoradionecrosis.

Tumor recurrence usually happens in the first two years; however, in 17% of the cases it happens after 6 years of evolution^6^^,^^9^^,^^15^, justifying patient follow up for at least 6 years^6^^,^^9^^,^^15^.

## FINAL REMARKS

The very rarity with which inverted papillomas affect the nasal cavities (bilateral) makes this report so important, especially considering post-operative follow up because, despite being a benign tumor, the inverted papilloma is a very aggressive tumor. Surgical treatment must be careful, with previous radiological study of tumor boundaries, so as to carry out the best technique and thus, remove it completely and reduce recurrence risks.
